# How to Apply FMT More Effectively, Conveniently and Flexible – A Comparison of FMT Methods

**DOI:** 10.3389/fcimb.2021.657320

**Published:** 2021-06-04

**Authors:** Adorján Varga, Béla Kocsis, Dávid Sipos, Péter Kása, Szabolcs Vigvári, Szilárd Pál, Fanni Dembrovszky, Kornélia Farkas, Zoltán Péterfi

**Affiliations:** ^1^Department of Medical Microbiology and Immunology, University of Pécs Clinical Centre, Pécs, Hungary; ^2^1^st^Department of Internal Medicine – Department of Infectology, University of Pécs Clinical Centre, Pécs, Hungary; ^3^Institute of Pharmaceutical Technology and Biopharmacy, University of Pécs Faculty of Pharmacy, Pécs, Hungary; ^4^Institute for Translational Medicine, University of Pécs Medical School, Pécs, Hungary; ^5^Institute of Bioanalysis, University of Pécs Medical School, Pécs, Hungary

**Keywords:** faecal microbiota transplant, lyophilisation, capsules, *Clostridioides difficile* infection, recurrence, faecal supernatant, faecal sediment

## Abstract

**Purpose:**

Metronidazol and vancomycin were long the two best options against *Clostridioides* (formerly *Clostridium*) *difficile* infections (CDI). Now, the cost of new drugs such as fidaxomicin directs us towards alternative treatment options, such as faecal microbiota transplant (FMT). Its effectiveness is similar to fidaxomicin. There are questions regarding its safety, but the biggest challenges are prejudice and inconvenience. Most protocols refer to FMT applied in the form of a solution. We investigated different modalities of FMT.

**Methods:**

Instead of using nasoenteric tubes or colonoscopy, we place frozen or lyophilised stool in non-coated, size “00”, hard gelatine capsules or enterosolvent, size “0” capsules.

**Results:**

We found that non-coated, size “00”, hard gelatine capsules are appropriate for conducting FMT. Capsules containing lyophilised supernatant with a low number of bacteria have been proven to be non-inferior to other FMT modalities. The primary cure rate in the supernatant group was 93.75%, and 66.67% in the sediment group. The overall cure rate was 82.14%. Depending on the protocol, 4–7 capsules are sufficient per patient. Capsules can be stored for up to one year at -20°C.

**Conclusions:**

FMT is a feasible alternative to antibiotic treatments in CDI. Our method makes the process flexible and less inconvenient to patients. Long storage time allows a consistent supply of capsules, while small volume and formulation make the procedure tolerable.

## Introduction

*Clostridioides difficile* infections remain a major public health issue. Estimating the burden of CDI is a challenging task, studies showed an annual cumulative incidence rate for all ages of 49.36 per 100 000 population (Balsells et al., 2019). From 2011 through 2017, the estimated national burden was 450000 cases in the United States (Guh et al., 2020), and approximately 120000 cases in the European Economic Area (ECDC, 2015). Depending on several factors (ribotype, age, health condition), the mortality varies between 4-7% (Kwon et al., 2015). Metronidazole and vancomycin are still in the guidelines, but neither offers a satisfying level of effectiveness (Schluger et al., 2019). Fidaxomicin is a successful antibiotic with respect to both effectiveness and recurrence rate (Madoff et al., 2019). A network meta-analysis concluded the cure rates and recurrence rates of the aforementioned antibiotics as follows: in severe CDI, metronidazole had a clinical cure rate of 76%, while the cure rate for vancomycin varied between 90% and 97%. Fidaxomicin was found non-inferior to Vancomycin (91,7% versus 90,6%, respectively) in that comparison, and showed significantly lower rates of recurrence 12.7% versus 26.9%, respectively) (Okumura et al., 2020). Faecal microbiota transplant (FMT) is considered a highly effective option in CDI (Orenstein and Patron, 2019) as well, besides being much more economical. The European Consensus Conference on FMT in clinical practice board has recommended FMT as treatment option for both mild and severe recurrent CDI (rCDI) (Cammarota et al., 2017). The strength of recommendation for the first episode of CDI was described as weak.

A major disadvantage of FMT is the asymmetry between its labour-intensiveness and the low case numbers: it is infrequently necessary, but when it is, it requires the constant presence of a healthcare professional. The conventional method consists of three major steps: finding and testing a donor, preparing the faecal filtrate and administering it to the patient. Finding a donor is often complicated, since many elderly patients have no adequate relatives, and if they have, healthcare professionals still have to deal with the prejudice of the potential donors towards the procedure. It is also a subject of debate whether or not relatives should be used as donors or whether pooled stool samples could be more effective (Barnes and Park, 2017). Stool banks could be highly beneficial in this regard, but they are currently not widespread at all (Cammarota et al., 2019). To improve flexibility, a number of studies have been done (Youngster et al., 2016; Youngster and Gerding, 2017; Cold et al., 2019; Vigvari et al., 2019a) or are currently in progress to find a feasible solution for sample storage in clinical practice. Conventional FMT methodology uses three types of administration: through a nasogastric (NG) or nasojejunal (NJ) tube or *via* colonoscopy. None of these methods can be considered convenient, and all of them are invasive procedures, though undoubtedly effective. Several studies have examined the differences between these three methods. One of them showed that the symptoms resolved within a day after NJ-FMT without any recurrence (in 16 cases), while they resolved within a day *via* NG tube in 88.64% of the cases, with a 11.36% recurrence rate observed (60 patients in total) (Vigvari et al., 2019b). An earlier study in the same hospital (with a lower case number: 15 NJ and 15 NG) showed comparable results: the primary cure rate was 100% in the NJ group, and 80% for the primary cure rate and 93.3% for the secondary cure rate in the NG group (Vigvari et al., 2014). Other studies showed no significant difference between the lower and upper gastrointestinal routes for administration (Goldenberg et al., 2018); their use depends on preference. However, in the case of colonoscopy, the increased risk of perforation due to inflammation of the colon has to be noted.

FMT capsules combine the advantages of antibiotics (convenience) and FMT (high efficacy and cost-efficiency). Clinical trials aimed to examine the frozen stool used (Youngster et al., 2016) or the lyophilised faecal matter (Youngster and Gerding, 2017) in capsules. These trials demonstrated a high efficacy in resolving recurrent CDI, comparable to the conventional applications. Finding the key components responsible for the beneficial effect is a highly researched topic (Elangovan et al., 2019; Saltzman et al., 2019; Péterfi and Vincze, 2020)], but it has not yielded any promising results so far.

Our hypothesis was that a faecal transplant can be carried out with greater effectiveness and more conveniently by using lyophilised stool placed in capsules than by administering faecal solution through a nasoenteric tube.

The primary endpoint was the discontinuation of diarrhoea, the secondary endpoint the cure and prevention of relapse within six months,

The tertiary endpoint was to elucidate if capsules containing lyophilised faecal supernatant only are more effective than capsules containing lyophilised faecal sediment only.

The main focus of the study was to develop an FMT method that

- results in a small volume of end product
- can be administered conveniently
- has better effectiveness than regular FMT modalities
- can be manufactured cost- and time-efficiently.

## Materials and Methods

### Study Design

We studied different FMT methods, focusing on encapsulated lyophilised faecal material. We aimed to develop different modalities of capsule making; therefore, we kept the first steps of the sample preparation and implemented additional steps to achieve a moderate, more tolerable amount of the end product.

**Figure 1:**
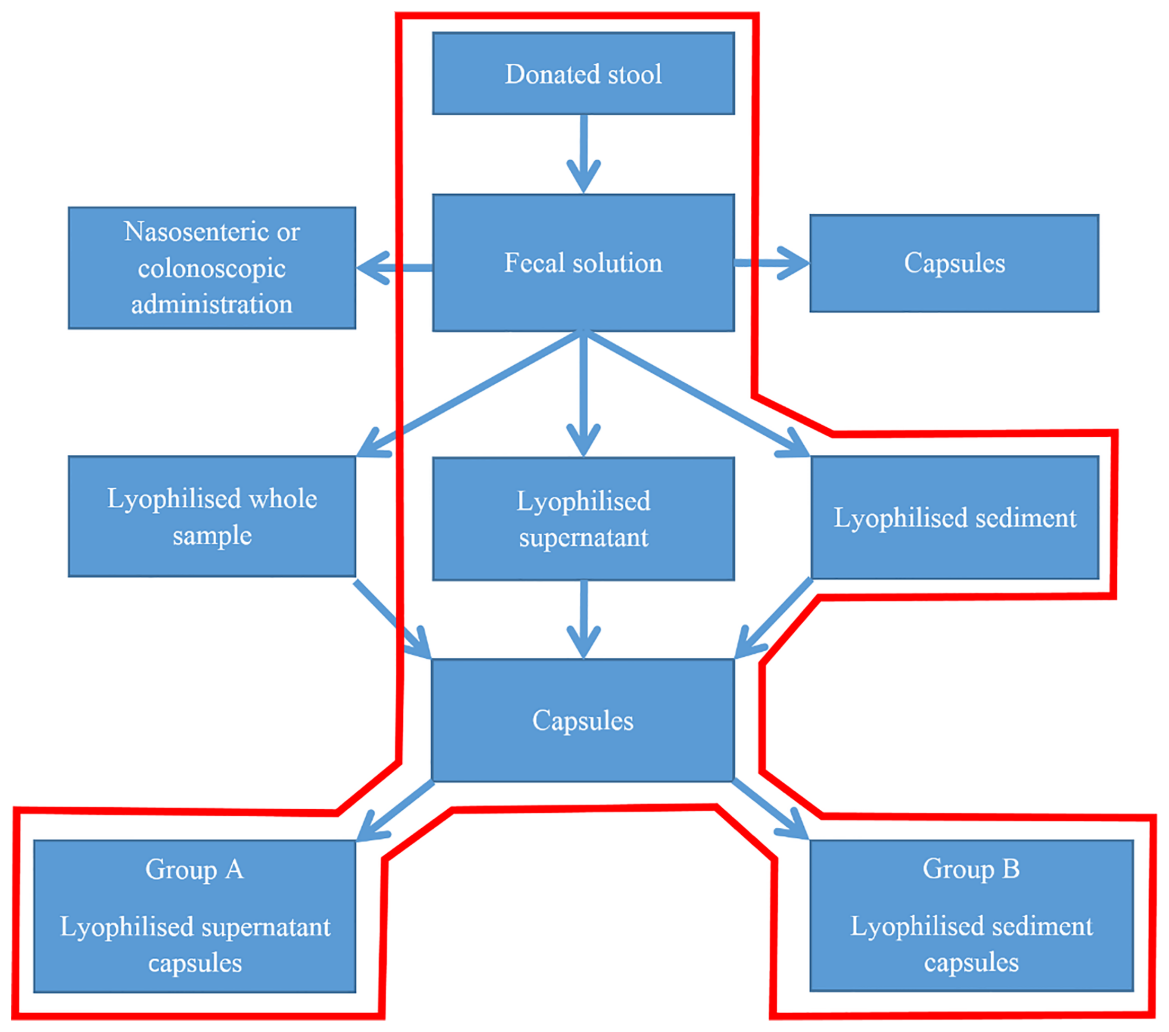
Flowchart of our study.

Our study had two arms (Group A and B). Patients in Group A were treated with encapsulated lyophilized faecal supernatant, while in Group B encapsulated lyophilized faecal sediment was administered (**Figure 1**).

### FMT Recipients

Patients with recurring *Clostridioides difficile* infection were enrolled with at least two recurrences. A recurrence was defined as an episode of multiple diarrhoea within six months after the last CDI.

### Donor Selection

For our study, the same donor was used in all the cases. The donor was a 27-year-old Caucasian male with normal BMI (body mass index). He was screened (history taking, blood tests and faecal pathogen testing) according to the international guidelines (Cammarota et al., 2017; Mullish et al., 2018; Bálint et al., 2020).

### Stool Collection

At the beginning of the study, we decided to use one donor for all our patients. This decision was made for multiple reasons. We wanted to exclude the bias that multiple donors would have caused. We also found this approach safer, since we were in constant contact. Another important aspect was the simplicity that this approach provided in terms of sample collection. The donor was tested according to the comprehensive guidelines, and donor stool was tested for vancomycin-resistant *Enterococci* (VRE) and multidrug resistant Gram negative microorganisms. Although the exact characteristics of the “optimal donor” are still unknown, there are international protocols for donor selection [**Tables 1** and **2** (Kim and Gluck, 2019)]. We provided specimen collection pans (Covidien) and sterile containers for the donor to facilitate sample collection. The donor transferred the whole stool sample into the container with a single use plastic spoon, then notified the contact person to organise the transport.

**Table 1:** Suggested exclusion criteria.

Age <18 years or >65 years
-Body Mass Index (BMI) >30kg/m2
-Metabolic syndrome
-Moderate to severe undernutrition
-History of antibiotics use in the last 6 months
-Diarrhea within the last 3-6 months
-History of *Clostridioides difficile* colitis
-Immune disorder or use of immunosuppressive medications
-History of drug use or other recent risk factor for HIV (Human Immunodeficiency Virus) or viral hepatitis
-History of travel to a tropical region in the last 3 months
-Any gastrointestinal illness (IBD, Inflammatory Bowel Disease; IBS, Irritable Bowel Syndrome, gastrointestinal malignancy, or major surgery) or complaints
-History of chronic pain syndrome (fibromyalgia, chronic fatigue syndrome)
-Neurologic or neurodevelopmental disorders
-History of malignancy

**Table 2:** Suggested laboratory tests for potential donors for faecal microbiota transplantation.

Tests	Blood	Stool
**Bacteria**	*Treponema* spp.	Enteric pathogen culture: *Salmonella, Shigella, Campylobacter* spp. *Helicobacter pylori –* EIA VRE antibiotic sensitivity test to prevent the use of stool containing polyresistant strains.
**Viruses**	Hepatitis A virus IgM Hepatitis B virus surface antigen Anti-hepatitis C virus HIV 1 and 2	Norovirus EIA or PCR Rotavirus EIA
**Parasites**	*Entamoeba histolytica* *Strongyloides stercoralis*	Ovum and parasite *Microsporidia* *Ghardia* faecal antigen/EIA *Cryptosporidium* EIA AFB for *Isospora* and *Cyclospora*
**Others**	Complete blood count Liver function test ESR and CRP	*Clostridium difficile* test PCR of toxin genes Others

AFB, Acid Fast Bacillus; CRP, C Reactive Protein; EIA, Enzyme Immuno Assay; ESR, Erythrocyte Sedimentation Rate; HIV, Human Immunodeficiency Virus; IgM, Immunoglobulin M; PCR, Polymerase Chain Reaction.

### Stool Preparation

The donated stool is processed within two hours of defecation. The stool samples were stored on room temperature until the begin of the processing. Reusable tools are sterilized with ethylene oxide. After adding 200 mL of sterile physiological saline solution (0.9% sodium chloride) to 60 g of the sample, the stool is homogenized with a household mixer (AEG HM 250). The initial solution is filtered with a household pasta sieve to eliminate debris. A secondary screening is carried out with another sieve with a finer mesh. Depending on the consistency of the faecal matter, the resulting volume is approximately 220 mL. This volume is then placed in 50 mL tubes (Sarstedt Inc., Nümbrecht, Germany) and centrifuged for 10 minutes at 827 g (MPW-380R, Poland) to eliminate the smaller debris. The slurry is discarded, and the supernatant is handled in 100 mL portions. With these steps, approximately 120 mL of macroscopically homogenous solution can be made from 60 g of initial stool sample. This procedure is carried out under laminar airflow in a dedicated cabinet on room temperature.

### Steps According to the Regular Protocol and Beyond

**Figure 2:**
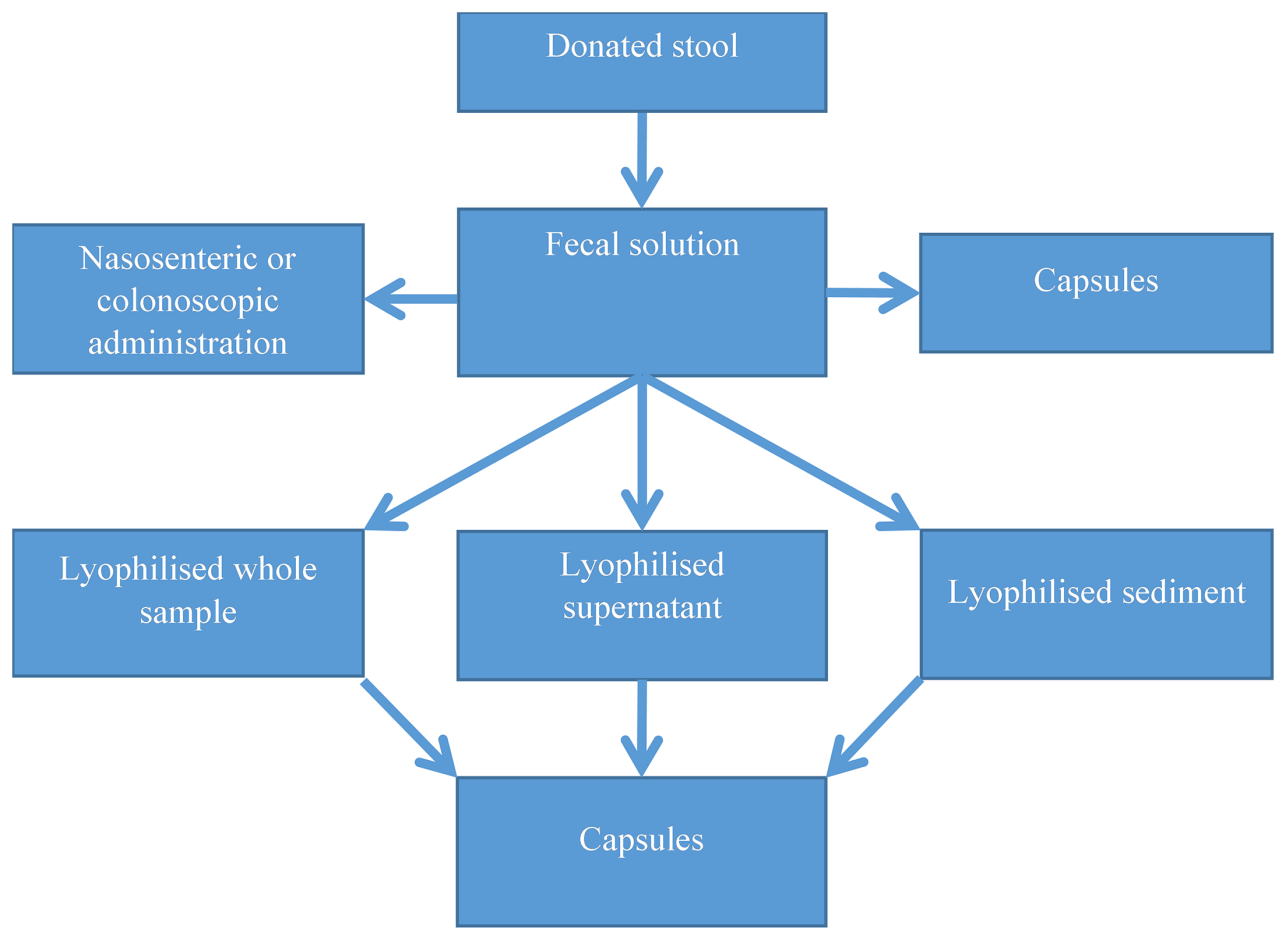
Options to administer the faecal solution. Lyophilised samples can be resuspended and administered *via* a nasoenteric tube or colonoscopy (not shown in the figure).

At this point, we have multiple options (**Figure 2**). We can either proceed with the administration of the faecal solution through a nasoenteric (NG or NJ) tube or through colonoscopy, freezing the faecal solution for later use, freeze-drying the 100 mL solution (full sample) or carry out a secondary centrifuge step to achieve a bacteria-rich sediment and a supernatant with significantly fewer bacteria. Yet another method is the use of frozen faecal material in capsules.

### Administration of the Fresh Faecal Solution Through a Nasoenteric (NG or NJ) Tube

Patients should not eat on the day of FMT. Premedication is administered (proton pump inhibitor and prokinetic). After insertion of the nasogastric tube, 100 mL of faecal solution is transferred with a syringe. In the case of the NJ tube, the syringe is loaded into an enteral feeding pump and the 100 mL volume is transferred in one hour, while the process takes ten minutes for the NG tube. Following the administration of the faecal material, 100 mL of fluid is placed in the syringe to clear the remaining material from the tube. After the procedure, patients are instructed to sit in a 45° upright position for two hours (Vigvari et al., 2019).

### Administration *via* Colonoscopy

Patient preparation is similar to that in diagnostic colonoscopy. Macrogol is administered, and patients should not eat solid food 24 hours prior to FMT. Loperamide is administered per os to aid the retention of the transplanted faecal material. The colonoscopy is performed with conscious sedation with fentanyl and midazolam, and the patients are placed in a right lateral recumbent position. The transplant material is injected into the proximal colon (into the coecum, if possible). The procedure is carried out within ten minutes. The patients are then instructed to maintain the position described for one hour (Cammarota et al., 2015).

### Freezing the Faecal Solution for Later Use

100 mL of the faecal solution is kept at -20°C until use. Based on our stability analysis (not described in our current publication) samples can be used up to six months after preparation. On the day of the procedure, the frozen sample is thawed in a 30–35°C water bath for 2 hours (Vigvari et al., 2015; Jorgensen et al., 2017; Kumar and Fischer, 2020). Afterwards, the solution can be administered as described above.

### Freeze-Drying of the 100 mL Solution (Full Sample)

In case we decide to work with the full sample, we divide the 100 mL volume into two 50 mL portions. This is done to facilitate freezing and to aid the freeze-drying process (to achieve a larger surface area). The samples are frozen in glass containers (5 cm in diameter) at -20°C and lyophilised (Freeze Dryer Heto Drywinner model DW1.0). The lyophilisation is carried out under the following conditions: -40°C, 4*10^-4^ mbar, 36 hours. The lyophilised sample is homogenised in a mortar with a pestle and placed in an appropriate number of capsules or stored in a glass container at -20°C until use.

In the latter case, the samples are resuspended in 100mL of sterile distilled water on the day of use and administered through a nasoenteric tube or colonoscopy, as described above.

### FMT With Capsules

#### Frozen Faecal Material in Capsules

It is also possible to use capsules for FMT without lyophilisation, as described by Youngster et al. (2016). This method involves a concentrated form of faecal material with 10% glycerol. Double-encapsulation in hypromellose capsules (Capsugel, Cambridge, MA, USA) is used to prevent disintegration from filling to freezing and from freezing to administration. Another option is to form a polymer layer on the inside of the capsules. We aimed to achieve this with two substances: Eudragit^®^ L 30 D-55 (Ph. Eur. Methacrylic Acid – Ethyl Acrylate Copolymer (1:1) Dispersion 30%) and Eudragit^®^ RS 30 D (Ph. Eur. 0.3% Sodium Laurilsulfate, 1.2% Polysorbate 80 – Aqueous dispersion with 30% dry substance.) The substances are placed in the bottom half of the capsules using a pipette, then removed instantly. The capsules are then allowed to dry for 24 hours.

#### Lyophilised Faecal Material (Whole) in Capsules

After homogenising the lyophilised faecal solution with a mortar and pestle, the sample is placed in an appropriate number of capsules with a commercial capsule-filling tool (Capsule Machine, Capsule Connection, LLC, Prescott, AR, USA). The capsules are stored at -20°C.

#### Non-Coated Capsules

These size “00”, hard gelatine capsules have no special coating and are filled with the capsule-filling tool noted above. Due to the lack of coating, they are prone to dissolve shortly after entering a wet environment (such as the stomach). In physiological saline solution (0.9% sodium chloride), they start to lose stiffness within a minute and fall apart after 15 minutes. In the presence of a pH level of 2, they dissolve within a minute.

#### Enterosolvent Capsules

Enterosolvent capsules (Vcaps^®^ Enteric Capsules, Capsugel, Cambridge, MA, USA) are used to compare the efficacy of the two capsule types. Since these capsules are size “0”, we need an appropriate model of the filling equipment from the same manufacturer (Capsule Machine, Capsule Connection, LLC, Prescott, AR, USA.) These capsules survive the acidic environment of the stomach and dissolve at around pH 5. Although they can sustain their integrity in an acidic environment, they also lose stiffness within one minute, just like the non-coated capsules.

#### Separating the Supernatant From the Sediment

In the other scenario, we centrifuge 100 mL at a higher g force (15 minutes at 3309 g) to separate the bacteria from the solution. The volume of the resulting bacteria-rich sediment is around 30 mL, which is resuspended in 10 mL of sterile physiological saline solution (0.9%) for ease of transfer to the containers. The supernatant is divided into two containers to aid the freeze-drying process. Freeze-drying and capsule filling occur under the same conditions and with the same procedure as above.

#### Clinical Trial With Capsules

Patients were randomised into two groups (A and B). Group A was treated with supernatant capsules, while Group B received sediment capsules. Because of the visible differences between the two treatments, neither the patients nor the physicians were blinded to the randomisation groups. The number of capsules varied due to the changes of the faecal material provided; it varied between 5 and 7 for the supernatant capsules and between 3 and 5 for the sediment capsules.

Patient preparation is similar to that used for the nasogastric tube protocol. Patients are instructed to swallow the capsules one by one within five minutes. They are also provided with fluid to aid them in swallowing. After the procedure, the patients are instructed to sit in a 45° upright position for two hours.

#### Statistical Analyses

The randomisation of the patients was tested based on age and sex. The Chi-squared test was used to compare the efficacy of the two methods (**Table 3**).

**Table 3:** Comparison of efficacy of supernatant and sediment capsules.

Supernatant/Sediment * Succes/Failure Crosstabulation					
			Success/Failure		Total
			Success	Failure	
Supernatant/Sediment	Supernatant	Count	15	1	16
		Expected Count	13,1	2,9	16,0
	Sediment	Count	8	4	12
		Expected Count	9,9	2,1	12,0
Total		Count	23	5	28
		Expected Count	23,0	5,0	28,0
**Chi-Square Tests**					
	Value	df	Asymp. Sig. (2-sided)	Exact Sig. (2-sided)	Exact Sig. (1-sided)
Pearson Chi-Square	3,429^a^	1	,064		
Continuity Correction^b^	1,831	1	,176		
Likelihood Ratio	3,519	1	,061		
Fisher’s Exact Test				,133	,089
Linear-by-Linear Association	3,307	1	,069		
N of Valid Cases	28				

^a^2 cells (50,0%) have expected count less than 5. The minimum expected count is 2,14.

^b^Computed only for a 2x2 table.

### Survival of the Bacteria

To assess the difference in the number of colony-forming units (CFU) between the supernatant and the sediment, we conducted (1) aerobic cultivation on blood agar, eosin-methylene blue agar, chocolate blood agar, chocolate blood agar plates with vancomycin (40 mg/L) and Sabouraud agar and (2) anaerobic cultivation on blood agar (Sigma - Aldrich Kft. Budapest, Hungary). CFU numbers were counted after 48 hours of incubation at 30°C. The incubation temperature was chosen, because 30°C is suitable for fungi as well as for bacteria. In total, we received ten stool samples from our donor. At the end of lyophilisation, portions of 10 mg were placed in Eppendorf tubes for storage. Cultivation was carried out at six different time points: end of lyophilisation, and two days, two weeks, one month, three months and six months after lyophilisation. Samples were stored at four different temperatures: +20°C, +4°C, -20°C and -80°C. Samples were resuspended to their initial concentration and diluted to make counting possible on the agar media. The following media were used: blood agar plates for aerobic and anaerobic cultivation, and eosin methylene blue agar, chocolate blood agar and chocolate blood agar with vancomycin and Sabouraud agar plates for aerobic cultivation. The CFU number was counted after 48 hours.

## Results

It has already been proven that FMT is more effective in CDI than metronidazole and vancomycin and has similar efficacy to fidaxomicin. So far, no major differences have been found between the FMT modalities. The efficacy through the NG route is around 80% for the primary cure rate and around 95% for the secondary cure rate (Vigvari et al., 2014). In the case of the NJ route, the primary cure rate may be 100% (Vigvari et al., 2019b). The primary cure rate for FMT *via* colonoscopy varies around 85%, while the secondary cure rate may exceed 97% (Allegretti et al., 2014).

The production of the FMT capsules takes 3–4 days, and a net of eight labour hours (in comparison, the conventional procedure – using a nasogastric or nasojejunal tube – takes around two hours and can be administered immediately).

The mean weight of the full dry sample (no secondary centrifuge step) is approximately 3 g and requires around seven size “00”, hard gelatine capsules (0.43 g/capsule). The lyophilised supernatant weighs an average of 1.8 g and can fill 4–6 capsules (0.38 g/capsule), while the sediment weighs approximately 2 g after lyophilisation and requires four of the regular capsules (0.33 g/capsule).

We found that capsules can be effectively at -20°C used even after one year of storage, although most of them were used up within six months.

**Figure 3:**
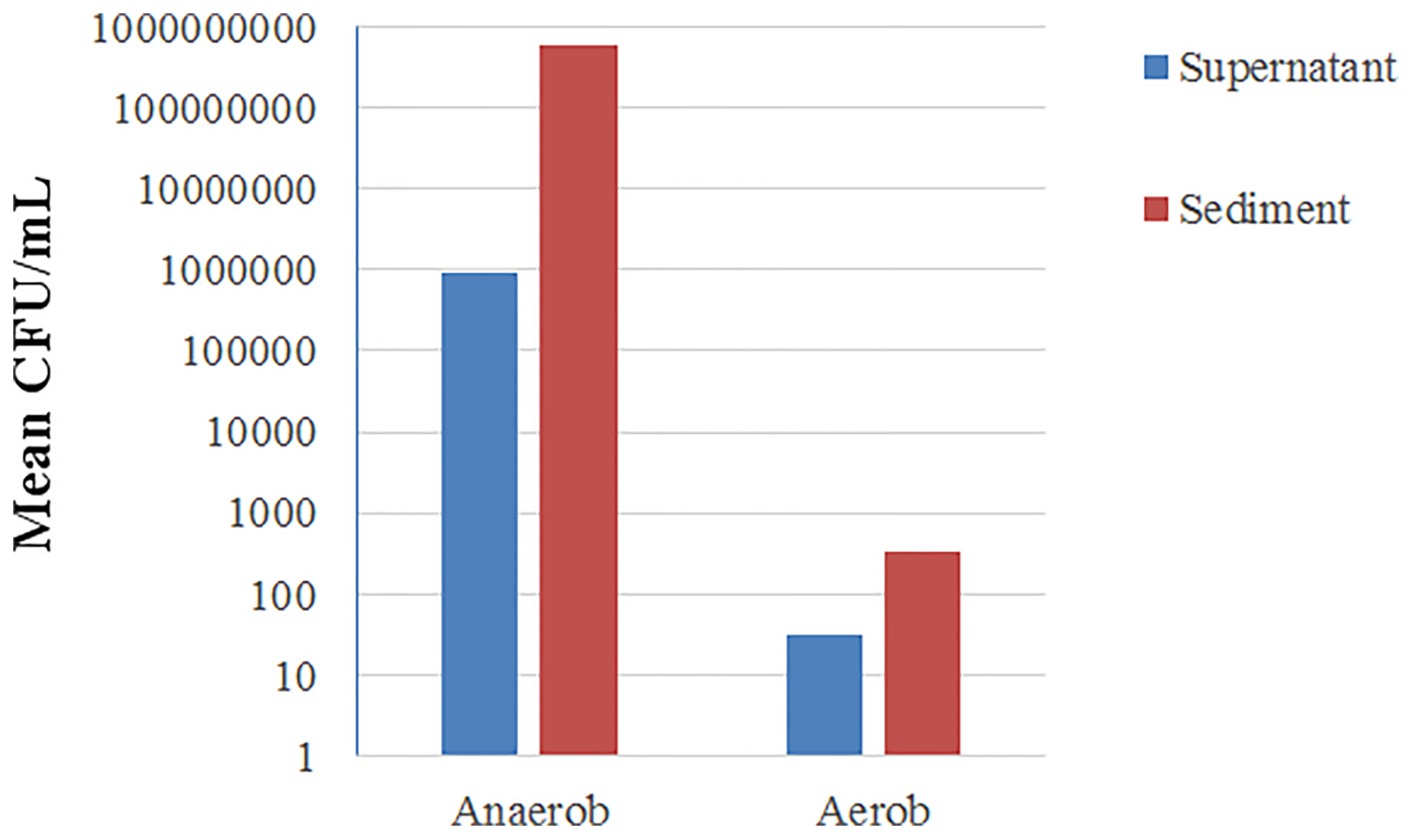
Mean CFU counts on a logarithmic scale in anaerobe and aerobe cultures from supernatant and sediment samples. CFU, Colony-Forming Units.

Our procedure did not result in a completely bacteria-free supernatant. Parallel aerobic and anaerobic cultures showed an approximately 600-fold difference in the CFU counts between the supernatant and the sediment samples (**Figure 3**). The remaining CFU count in the supernatant was 5*10^6^ CFU/mL on average (3*10^9^ CFU/mL in the sediment).

In respect of the enterosolvent capsules, due to their smaller size, the number of capsules required was higher. From the full dry sample, 0.29 g fit into a single capsule, while it was 0.26 g for the supernatant and 0.23 g for the sediment, resulting in a 45% increase in the number of capsules required.

In the case of frozen faecal material in capsules, starting with 60 g of initial faeces, 40 capsules had to be administered to one patient. Using the Eudragit^®^ solutions, we found that the capsules suffer slight deformation during the film-forming procedure but can be closed properly after drying. The extent of deformation can be moderated by facilitating the initial drying process with a hair dryer. In the case of Eudragit^®^ L 30 D-55, the capsules held their stiffness for 60 minutes after contact with aqueous solutions, while Eudragit^®^ RS 30 D capsules started to lose stiffness after five minutes.

### Clinical Results

From January 2018 to December 2019, 28 patients were randomly enrolled in the two groups (Female: 17; Male: 11; mean age: 68.8; age range: 35-83) In Group A, 16 subjects (Female: 10; Male: 6; mean age: 64.79) received capsules that contained lyophilized supernatant. In Group B 12 subjects (Female: 7; Male: 5; mean age: 66.47; age range: 43 - 82) were administered capsules that contained lyophilized sediment. The median of the recurrences was 2, the range was 2-4 in both groups. No patients refused the treatments.

**Table 4** summarizes the strengths and weaknesses of the examined FMT methods.

**Table 4:** Analysis of administration modalities for faecal transplant.

Method	Strength	Weakness
**Fresh faecal solution through nasogastric tube**	- Preparation is easier and - takes less time	- Invasive - uncomfortable for the patient - storage is a problem
**Local “stool bank” from frozen faecal solution**	- more flexible than fresh FMT	- has to be thawed before use
**Freeze-drying the full sample**	- lyophilisation provides longer storage time and - lower storage requirements no secondary centrifuge step is needed - convenient to the patients	- lyophilisation adds 36 hours to the sample preparation time - larger volume than the sediment/supernatant
**Freeze-dried supernatant**	- low volume convenient to the patients - lyophilisation provides longer storage time and - lower storage requirements	- time consuming - secondary centrifuge step needed
**Freeze-dried sediment**	- low volume - convenient to the patients - lyophilisation provides longer storage time and - lower storage requirements	- time consuming - secondary centrifuge step needed
**Hard gelatine capsules**	- low volume - convenient to the patients - lyophilisation provides longer storage time and - lower storage requirements	- time consuming
**Enterosolvent capsules**	- convenient to the patients - lyophilisation provides longer storage time and - lower storage requirements - can provide better efficacy?	- time consuming - due to the smaller capsule size, more capsules are necessary than from hard gelatine capsules - special capsules
**Frozen FMT capsules**	- convenient to the patients - faster preparation	- larger volume - special capsules

FMT, Faecal Microbiota Transplantation.

### Study Outcomes

Group A

In the supernatant group, 15 of the 16 patients (93.75%) were cured after a single portion of capsules. Two of them experienced a relapse shortly after transplantation (within three weeks) and were cured after fidaxomicin therapy. The only patient in the non-successful group had already experienced 22 recurrences beforehand. One patient from the successful group underwent a colectomy due to IBD later on. One of the patients has passed away due to a non-related disease since then.

Group B

In the sediment group, eight of the twelve (66.67%) patients were cured after a single portion of capsules. None of them experienced a relapse within six months. One of the cured patients remained *Clostridioides difficile* toxin A-positive without clinical symptoms. Her stool became negative after a following fidaxomicin therapy. Two patients were resistant to any other therapy, with FMT being effective in one of them and the other patient being cured with an additional fidaxomicin therapy. One patient has passed away due to a non-related disease since then.

The overall cure rate was 23/28 (82.14%). A summary of the results can be seen on **Table 5**.

**Table 5:** Primary results in Group A and B.

	Successful	Non-successful	Total
Group A	15	1	16
Group B	8	4	12

### Survival of Bacteria

**Figure 4:**
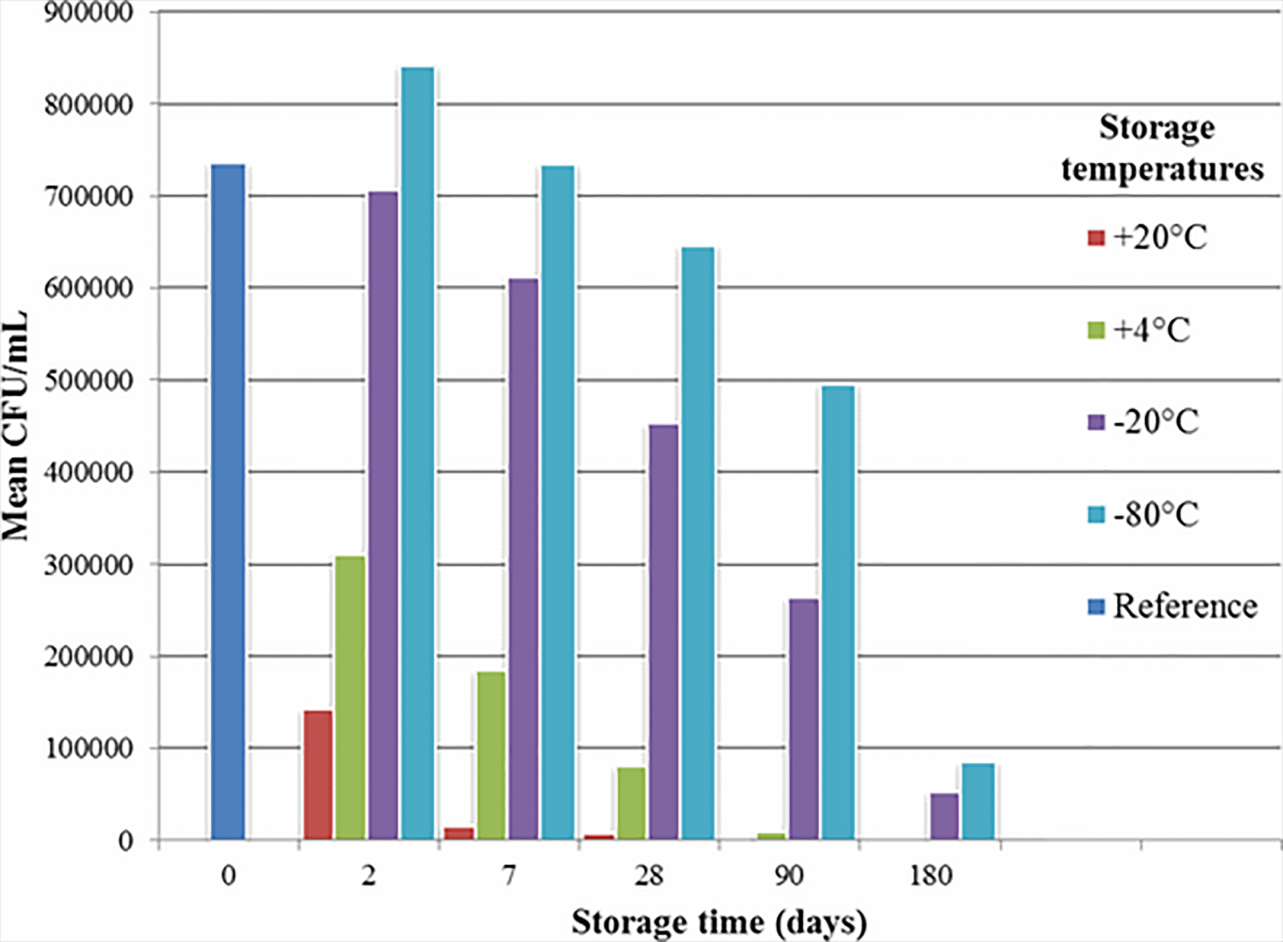
Survival of bacteria depending on the temperature of storage. CFU, Colony-Forming Units.

The observed survival rate of the bacteria can be seen in **Figure 4**. The initial concentration (“reference” in **Figure 4**) was 3*10^9^ CFU/mL, with 9*10^5^ CFU/mL at the end of the lyophilisation. The CFU’s represent the mean results of several experiments, aerobes and anaerobes combined.

## Discussion

Our results can be interpreted as proof of concept. However, this method is more labour-intensive and time-consuming than both the regular FMT method and the antibiotic treatment options. On the other hand, capsules have major advantages compared to all the other options. Numerous studies (Vigvari et al., 2014; Mathias et al., 2019) have already proven the superiority of FMT to metronidazole and vancomycin, while other studies (Madoff et al., 2019) have shown that FMT is non-inferior to fidaxomicin. Since capsule FMT has results that are similar to conventional FMT methods, these statements apply to our new protocol as well. A major advantage of capsule FMT to conventional FMT is the significantly lower volume: while 100 mL of faecal solution has to be administered through a nasogastric or nasojejunal tube, the same effect can be achieved with 4–7 capsules (depending on capsule type). This means lower storage space requirements and higher patient compliance.

Since stool has a very complex composition, the important components are yet unknown. We are planning further studies to elucidate the mechanisms behind the observed effect of FMT in CDI and ulcerative colitis. An earlier study (Ott et al., 2017) proved that sterile faecal filtrate was effective in CDI. This method results in a bacteria-free solution by filtration. On the one hand, this procedure seems less complicated than ours and clearly implies the importance of the non-bacterial elements of the faeces in CDI. On the other hand, however, clinical practice requires us to keep procedures as simple as possible. We do not need a completely bacteria-free solution, and while filtration seems easier, centrifuging may be a more accessible and therefore more viable option in FMT centres than stockpiling single-use filters. In regions with low financial resources, disposable equipment is less affordable than re-usable accessories and tools. Both this study and our findings with the supernatant capsules suggest that the key element might not only be the gut bacteria, but the presence of bacteriophages, different metabolites of bacteria, viruses, a specific composition of these contributors of the microbiome, or enzymes and antibodies of gastrointestinal fluid.

As for storage, -80°C might work better, but requires expensive equipment that may not be present on site. Our goal was to develop a widely accessible method. Glycerol may improve the stability of the samples as well, but it would also mean additional steps in the workflow – which we wanted to keep as simple as possible.

We used the same donor throughout our study, while others suggest that pooled samples (Barnes and Park, 2017) or faeces from relatives might be more beneficial. Our single-donor approach was not due to the universal donor theory, but to facilitate a comparison of the results among the recipients. Since we were therefore able to remain in contact with the donor, we also found this approach safer and we were not forced to constantly find new donors.

Enterosolvent capsules can be advantageous due to their ability to transport the faecal matter to the small intestines, thus protecting it from gastric acid. During the FMT procedure, a proton pump inhibitor (PPI) is administered to the patients as premedication, so the gastric juice can exceed pH 5 (Akiyama et al., 2019). This means that the faecal material is protected against gastric acid by PPI; on the other hand, PPI might be contraindicated in the case of enterosolvent capsules.

In respect of frozen capsules, we might be able to rapidly freeze the capsules after filling, but we would face problems by the bedside; patients would be required to swallow the frozen capsules within a very brief time frame. Another drawback of the frozen faecal material is the large volume. While a size “00”, hard gelatine capsule can contain 900 µL of material, this volume is only 600 µL for size “0” Vcaps^®^ Enteric Capsules. Therefore an excessive number of capsules is necessary (Youngster et al., 2016). In relation to the Eudragit^®^ solutions, L 30 D-55 seems the more suitable choice. In practice, five minutes is sufficient to fill 24 capsules (this is the size of our capsule-making tool) and close them, and they can also be frozen before the inoculum starts to leak. These solutions may also render the capsules resistant to gastric acid (they dissolve at pH 5.5), but since we only treat the bottom half of the capsules, this feature does not play a role. The top halves of the capsules could also be coated with this method, but the deformation of the capsules would prevent proper closing. We also noticed a slight decrease in capsule volume during the procedure, potentially resulting in an increase in the number of capsules required.

We achieved promising results by using non-coated capsules filled with lyophilised supernatant (with a 93.75% primary cure rate). Despite the higher number of CFU, the non-coated capsules filled with lyophilised sediment showed lower efficacy (with a 66.67% primary cure rate and a 72% overall cure rate). None of our patients mentioned discomfort in relation to the method, and no adverse events were observed.

### Conclusions

Irrespective of the exact modality, FMT is a feasible alternative to antibiotic treatments in CDI compared to nasoenteric or colonoscopic administration.

Our overall cure rate of 82.14% was lower than those of conventional FMT modalities.

We found a higher cure rate in the supernatant group (93.75%) than in the sediment group (66.67%), although the difference was not significant. We conclude that, rather than FMT directly requiring live intact bacteria for its efficacy, it was instead likely that one or more soluble factors associated with bacteria within the filtrate potentially mediated its mechanism of action.

Potential factors could be the bacteriophages (Caudovirales) or the metabonomics, especially short-chain fatty acids (SCFAs) (Segal et al., 2020). A study showed a higher success rate of FMT with donor samples containing higher fraction of *Caudovirales* within the stool virome, and after FMT the abundance of the order of *Caudovirales* bacteriophages reduced significantly in the recipient’s stool (Zuo et al., 2018).

Metabonomics is defined as “the quantitative measurement over time of the metabolic responses of an individual or population to drug treatment or other intervention” (Holmes et al., 2008). A well-studied group of metabolites SCFAs, these are products of the bacterial fermentation. Studies suggest, that SCFA producers may play an important role in the homeostatic balance in the microbiome (Atarashi et al., 2013; Paramsothy et al., 2017; Parada Venegas et al., 2019).

Due to the small volume and the formulation, patients found our method much more tolerable and convenient than the nasoenteric administration. When using lyophilised faecal material, the long storage time allows us to maintain a consistent supply of capsules even with small-scale manufacturing, making the method more flexible.

Non-coated, size “00”, hard gelatine capsules are sufficient for the effect. On the other hand, due to the relatively low case number and the labour-intensive procedure, the production of the preparations can be difficult to arrange. Simplifying FMT and making it more flexible may support research into alternative uses for the process.

Although it was effective to use the same donor for all our patients, it is yet to be elucidated if pooled stool is more beneficial. Our success with the low CFU number capsules calls the importance of bacteria in the effect of FMT against CDI into question.

## Data Availability Statement

The raw data supporting the conclusions of this article will be made available by the authors, without undue reservation.

## Ethics Statement

The studies involving human participants were reviewed and approved by Hungarian Medical Research Council (ETT-TUKEB, Budapest, Hungary. Approval number: IF-7762-4/2015). The patients/participants provided their written informed consent to participate in this study.

## Author Contributions

AV: Corresponding author, sample preparation, capsule making, data analysis. BK: Design and revision of the work. DS: Acquisition of data, development of the method (FMT). PK: Development of the method (capsules). SV: Development of the method (FMT). SP: Development of the method (capsules). NF: Data analysis, interpretation of data. FD: capsule making, revision of the work. ZP: Design and revision of the work, development of the method (FMT). All authors contributed to the article and approved the submitted version.

## Funding

Publication fees were granted by the University of Pécs Medical School (Hungary) (EFOP-3.6.3-VEKOP-16-2017-00009).

## Conflict of Interest

The authors declare that the research was conducted in the absence of any commercial or financial relationships that could be construed as a potential conflict of interest.

## Abbreviations

BMI, body mass index; CDI, *Clostridioides difficile* infection; CFU, colony-forming units; FMT, faecal microbiota transplantation; IBD, inflammatory bowel disease; NG, nasogastric; NJ, nasojejunal; PPI, proton pump inhibitor; VRE, vancomycin-resistant *Enterococci*.
